# Distribution of toxic metals and relative toxicity of airborne PM_2.5_ in Puerto Rico

**DOI:** 10.1007/s11356-020-11673-4

**Published:** 2021-01-02

**Authors:** Héctor Jirau-Colón, Jannette Toro-Heredia, Josué Layuno, Enrique Dionisio Calderon, Adriana Gioda, Braulio D. Jiménez-Vélez

**Affiliations:** 1 School of Medicine, Department of Biochemistry, University of Puerto Rico Medical Sciences Campus, Main Bldg. 2nd Floor B210, San Juan, 00936 Puerto Rico; 2 Center for Environment and Toxicological Research, San Juan, Puerto Rico; 3 Universidad Ana G. Méndez de Gurabo, Gurabo, Puerto Rico; 4 Pontifícia Universidade Católica do Rio de Janeiro, Rio de Janeiro, Brazil

**Keywords:** Environmental toxicology, Environmental pollution, Particulate matter 2.5, Toxic metals, Puerto Rico, Air pollution

## Abstract

The exposure to airborne particulate matter (PM) and its constituents is an important factor to be considered when evaluating their potential health risk. Transition metals found in PM are known to contribute significantly to the exacerbation of respiratory ailments. Exposure to these constituents results in the induction of oxidative stress in the bronchial epithelium, thus promoting the secretion of inflammatory mediators. Therefore, it is important to know the contributions of PM_2.5_ constituents to further investigate their relationship with toxic responses and associated health risks. PM_2.5_ samples from three rural (Humacao, Guayama, and Guayanilla) and two urban (more populated) sites (Bayamón and Ponce) from Puerto Rico were analyzed for various inorganic constituents. A total of 59 trace elements were analyzed, of which eight were considered with the greatest toxic potential. The highest annual average concentration of PM_2.5_ was reported at the urban site of Ponce (5.82 ± 1.40 μg m^−3^), while Bayamón’s average concentration was not as high (4.69 ± 1.30 μg m^−3^) compared to concentrations at the rural sites Humacao, Guayama, and Guayanilla (4.33 ± 1.20 μg m^−3^, 4.93 ± 1.50 μg m^−3^, and 4.88 ± 1.20 μg m^−3^ respectively. The concentration at the Ponce site exhibited the highest summer value (7.57 μg m^−3^) compared to that of all the rural sites (~ 6.40 μg m^−3^). The lowest summer PM_2.5_ values were obtained at the Humacao site with an average of 5.76 μg m^−3^. Average Cu and Zn concentrations were 3- and 2-fold higher at the urban sites (0.68 ng m^−3^ and 6.74 ng m^−3^ respectively) compared to the rural sites (0.17 ng m^−3^ and 4.11 ng m^−3^). Relative toxicity of inorganic PM extract indicates Bayamón (urban) and Guayama with similar low LC_50_ followed by Humacao, Guayanilla, and finally Ponce (urban) with the highest LC_50_. Of the eight potential toxic metals considered, only Fe was found to be higher at the rural sites. To our understanding, there are different sources of emission for these metals which potentially indicate main anthropogenic sources, together with the trade winds adding periodically volcanic and African Dust Storm particulates that affect Puerto Rico. These results are the first of their kind to be reported in Puerto Rico.

## Introduction

Airborne particulate matter (PM_2.5_), also referred to as particle pollution (USEPA 2016), is a complex mixture of extremely small particles and liquid droplets. The emergence of these particles due to the industrial revolution has made the present atmosphere quite different from what it was originally. Industrialization, urbanization, and population activity aggregates lead to an increase in anthropogenic emissions from both fossil fuel and biomass combustion (Kim et al. 2015). Three different types of ambient particles are defined by size: ultrafine (less than 0.10 μm), fine (0.10–2.5 μm), and coarse (2.5–10 μm). Fine particulate matter (PM_2.5_) originates from fossil fuel combustion, road traffic, agriculture, and industrial manufacturing processes (Andreauet al. 2012; Li et al. 2008). It has been well-established that PM_2.5_ can penetrate deeply into the lung system and irritate, corrode, and cross the alveolar wall, affecting and impairing lung function (Guan et al. 2016).

PM fractions are known to contain organic materials (e.g., bacterial endotoxins), fungi (spores), pollen fragments, polycyclic aromatic hydrocarbons (PAH), and carbonaceous materials. Furthermore, inorganic materials like toxic metals, minerals (quartz, silicates), salts (ammonium sulfates and nitrates), and soil dust particles are also part of airborne PM complex mixture (Bell and Ebisu 2012; Dreher et al. 1997; Molinelli et al. 2006; Schlesinger et al. 2006; Ying et al. 2009). Some of these toxic metals are elements with high densities, specific gravities, and/or atomic weights. Their source of entry varies among deposition of atmospheric particulates and disposal of metal-enriched sewage sludge and effluents, as well as byproducts from metal mining processes (Srivastava and Majumder 2008). Resuspension of roadside dust and soil contributes as another potential source of the toxic metals found in PM. Of these contaminants, Iron (Fe) is a metal with significant concentrations in air pollution particles of most sources of emission, while lead (Pb) is mostly linked to vehicles, resuspended soil, and oil-burning sources (Schroeder et al. 1987).

Based on the work of Tirado-Delgado, since 1995, the Soufrière Hills volcano eruptions contributed much of the natural PM input in Puerto Rico (Tirado-Delgado 2009). This is a natural source of particle pollution for the Caribbean Islands contributing to the toxic metal exposure. The following toxic metals have been reported in volcanic ash from Soufrière Hills: chromium (Cr, 477.00), lead (Pb, 74.79), nickel (Ni, 108.90), cadmium (Cd, 231.00), cobalt (Co, 14.24), copper (Cu, 69.70), zinc (Zn, 57,775.00), arsenic (As, 21.40), mercury (Hg, 0.04), selenium (Se, 2.88), and tin (Sn, 7.80) all in nanogram per cubic meter (Allen et al. 2000). Nonetheless, atmospheric deposition is considered to be the major source of toxic metals such as Hg, Cd, Pb, and others to various ecosystems, thus becoming a global environmental pollution problem. It is widely known that the concentration range of particulate matter and atmospheric pollutants varies between toxic metal, just as shown by Schroeder et al. in his work (1987).

The toxic metals previously discussed are considered some of the most serious pollutants in the environment, known to be persistent and to bioaccumulate (Gleyzes et al. 2002). When inhaled, a complex mixture of metals and organics may induce allergies and cause or exacerbate asthma, cardiovascular diseases, and, in extreme cases, lung or kidney and bladder cancer or mortality, with a marked effect on an increase of over 10.00 μg m^−3^ (Turner et al. 2011, 2017).

In this study, we evaluate and compare particle pollution in two main urban cities of Puerto Rico, one on the north coast (Bayamón) and one on the southern (Ponce). We also evaluate PM_2.5_ and toxic metals in three rural cities from the south coast (Humacao, Guayama, and Guayanilla). This type of study and information has neither been previously reported nor has been a comparison done between rural and urban sites on the island. With the current understanding of the importance of PM metal toxicity, we must further explore the presence of these constituents, and fill the gap in the understanding of the etiology and source of exposure against the Puerto Rican population.

## Materials and methods

**Fig. 1:**
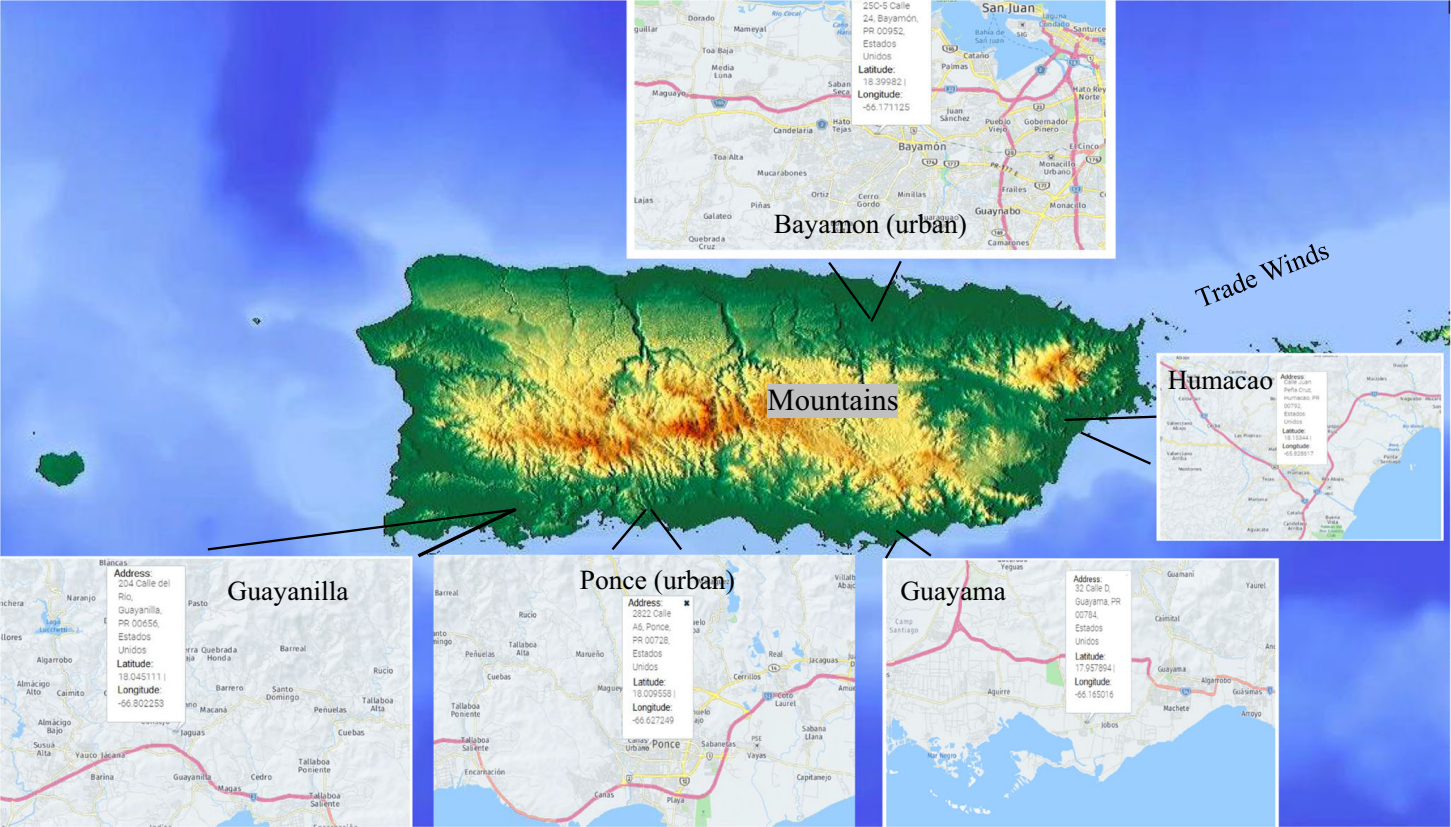
The island of Puerto Rico, illustrating the five sampling stations in their respective municipalities, urban (Ponce and Bayamón), and rural (Humacao, Guayama, and Guayanilla). Trade wind direction is from north to east. Bayamón is downwind from San Juan, which is also considered an urban area, while Guayama, Ponce, and Guayanilla are downwind from Humacao. Urban stations are marked on the map

This is the first time that levels of toxic metals in PM_2.5_ have been measured and reported in a systematic way around the island of Puerto Rico (Fig. 1), understanding, however, that there are significant weather differences between the north and south coast of Puerto Rico. The south coast reflects an arid environment with a northern mountain range barrier, while the north is more humid receiving the moisture of the northeastern trade winds. This is due to the topography of the island (with an east to the west-central mountain range) retaining humidity from the trade winds on the northern coast. The northern coast of Puerto Rico has historically been perceived as a more polluted environment by its residents. This stems from the presence of toxic industrial activity and vehicular traffic.

### Collection methods and measurement techniques

PM_2.5_ samples for the year 2013 were collected using a Partisol-Plus Model 2025 Sequential Air Sampler (Rupprecht and Patashnick Co., Inc., Albany, NY) on PTFE 2.0-μm Teflon filters by the Puerto Rico Environmental Quality Board (PR-EQB), which maintains a PM monitoring stations network at various locations in the Island as part of their monitoring program (Fig. 1). The air sampler maintains a flow rate of 1 m^3^/h through a single filter. Dates for sample selection were classified into three different groups, each covering 4 months, starting from January to April (S1), May to August (S2), and September to December (S3) for Bayamon and Humacao. For the other stations (Guayanilla, Ponce and Guayama) two period were included, Jan-May and August to December. S2 was of major interest given that it is associated with the passing of African dust events which are seasonal and enriched particulate matter with both organic and inorganic compounds (Jimenez-Velez et al. 2009). The sampling sites for this study were the following urban sites: Ponce (*n* = 99 filters) and Bayamón (*n* = 44 filters); and the rural stations: Humacao (*n* = 43 filters), Guayama (*n* = 94 filters), and Guayanilla (*n* = 94 filters). These sites can be observed in Fig. 1 and were selected based on location (urban or rural) and availability of the samples at the time. Filters from all the stations were collected throughout the year 2013 and stored at a temperature of 4° C. The PR-EQB was in charge of and kept custody of the samples before the commencement of this project. The storage following the collection of the samples was done following their established EPA guidelines. Samples were afterward refrigerated in our facilities for further analysis.

### Particulate Matter Extract preparation

All PM_2.5_ filters obtained from the PR-EQB were soaked in 50 mL of hexane/acetone 1:1 ratio and extracted using a microwave extraction apparatus (MAE) (Ethos Plus Microwave Labstation, Milestone Inc., Monroe, CT). Extraction glassware was washed using a modified cleanup procedure from a previous study (Molinelli et al. 2006). Briefly, four Teflon filters were introduced into the MAE Teflon closed vessel and soaked in approximately 50 mL of hexane/acetone solvent solution. The ID labels from each filter were removed by cutting it from the filter and were not included in the microwave extraction. MAE extraction was performed for 30 min; the program consisted of 5 min of heating the closed vessel to 80 °C and emission of microwaves at a power of 300 Oms. Extracted filters were then removed and other filters added to the remaining extraction solvent solution. The final resulting solution was combined in a 50-mL flask and left evaporating in a chemical hood under a slow and gentle nitrogen stream. As the solvent extract was evaporated, it was transferred to a smaller pre-weighed amber vial (at constant weight) until evaporated towards complete dryness. All extracts were stored at − 20 °C until further use. There was a ± 0.0001-g variation in sample weight after a complete evaporation was achieved. Efficiency ranges covering all the MAE extractions were approximately 39.49–54.88% of the fine fraction of the PM_2.5_ samples which accounts for almost half of the mass found on the filters.

### Elemental analyses of PM_2.5_ extracts

Dried samples of 0.10 to 1.00 mg were sent out for elemental analyses using an ICP-MS (ELAN DRC II 6000, Perkin Elmer). Each sample was diluted in 100 μL of bi-distilled nitric acid (HNO_3_, Merck, PA) and heated at 90 °C for 2 h to minimize polyatomic ion interferences. The final volume of extracts was completed to 2.50 mL with Nanopure water (Milli-Q, Millipore, USA). The element concentration was determined for all samples after two readings of 5 replicates each (10 readings per sample of which the instrument discards 3 and the average obtained from the remaining). Analyses included the following elements: silver (Ag), aluminum (Al), arsenic (As), gold (Au), barium (Ba), beryllium (Be), bismuth (Bi), bromide (Br), calcium (Ca), cerium (Ce), cesium (Cs), cadmium (Cd), cobalt (Co), chromium (Cr), copper (Cu), dysprosium (Dy), europium (Eu), erbium (Er), iron (Fe), gallium (Ga), gadolinium (Gd), germanium (Ge), mercury (Hg), holmium (Ho), lanthanum (La), lithium (Li), lutetium (Lu), potassium (K), magnesium (Mg), manganese (Mn), molybdenum (Mo), sodium (Na), nickel (Ni), neodymium (Nd), niobium (Nb), praseodymium (Pr), lead (Pb), rubidium (Rb), rhenium (Re), sulfur (S), antimony (Sb), scandium (Sc), selenium (Se), silicon (Si), samarium (Sm), tin (Sn), strontium (Sr), tantalum (Ta), terbium (Tb),thorium (Th), titanium (Ti), thallium (Tl), thulium (Tm), uranium (U), vanadium (V), tungsten (W), yttrium (Y), ytterbium (Yb), zinc (Zn), and zirconium (Zr). Analytical curves were prepared using PE5 and PE29 standard multi-element stock solutions (Perkin Elmer, USA), in a 5% v/v HNO_3_ solution. An Rh solution (40 μg L^−1^ in 1% v/v HNO_3_) was used as an internal standard, injected online with every solution.

### Cell cytotoxicity assay

The cytotoxicity of PM_2.5_ inorganic extracts was obtained employing the MTT Assay (ab211091, Abcam, Cambridge, MA, USA) following the manufacturer’s procedure on human lung cells (BEAS-2B). Briefly, an average of 10^4^ cells was seeded per well in a 96-well plate and left adhering for 24 h, then treated with PM_2.5_ extract for another 24 h at a concentration range of 25 to 200 μg/mL in cell media. After incubation, each well was carefully aspirated to remove treatment media and the MTT (3-(4,5-dimethyl thiazol-2-yl)-2,5-diphenyl tetrazolium bromide) reagent added at 50 μL of media + 50 μL/MTT Reagent per well. The plates were incubated for 3 h at 37 °C, aspirated carefully to remove the MTT reagent containing media, and then 150 μL of MTT solubilizing solution added to each well. The plate was placed on a shaker gently and left for 15 min to facilitate the solubilization of the formed formazan salts. The absorbance was measured at near 570 nm using a microplate reader.

### Statistical analysis

Descriptive statistics for toxic metal distribution by season and sampling sites were performed using the Minitab 17 Statistical Software (Minitab, Inc., State College, PA, USA). The statistical analysis to assess the relationship between sampling site PM_2.5_ concentrations was carried out using Student’s *t* test for paired comparisons between municipalities (statistically significant differences (CI: 95%, *p* < 0.05)). The toxic metal distribution and sampling site comparison graphs were generated and plotted using GraphPad Prism 8.0.0 for Windows (GraphPad Software, San Diego, CA, USA). The determination of the LC_50_ for the airborne PM_2.5_ graphs was generated using the ATT Bioquest LC_50_ Calculator (ATT Bioquest, Inc. 2020).

## Results

### Sampling site description and PM_2.5_ concentration

**Fig. 2:**
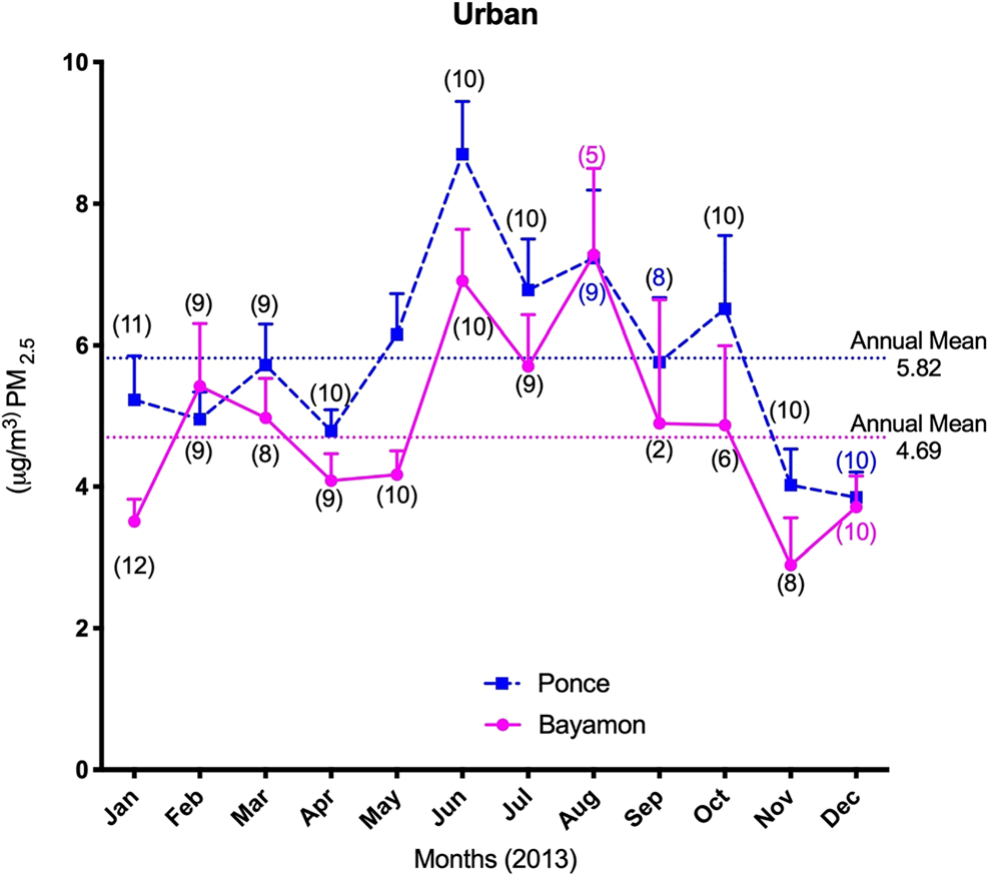
Average monthly concentrations and annual means of PM_2.5_ for urban sites Ponce and Bayamón during 2013. The number of PM_2.5_ measurements is shown in parenthesis under or over each mean monthly value. Error bars are shown as the standard deviation of each month

**Fig. 3:**
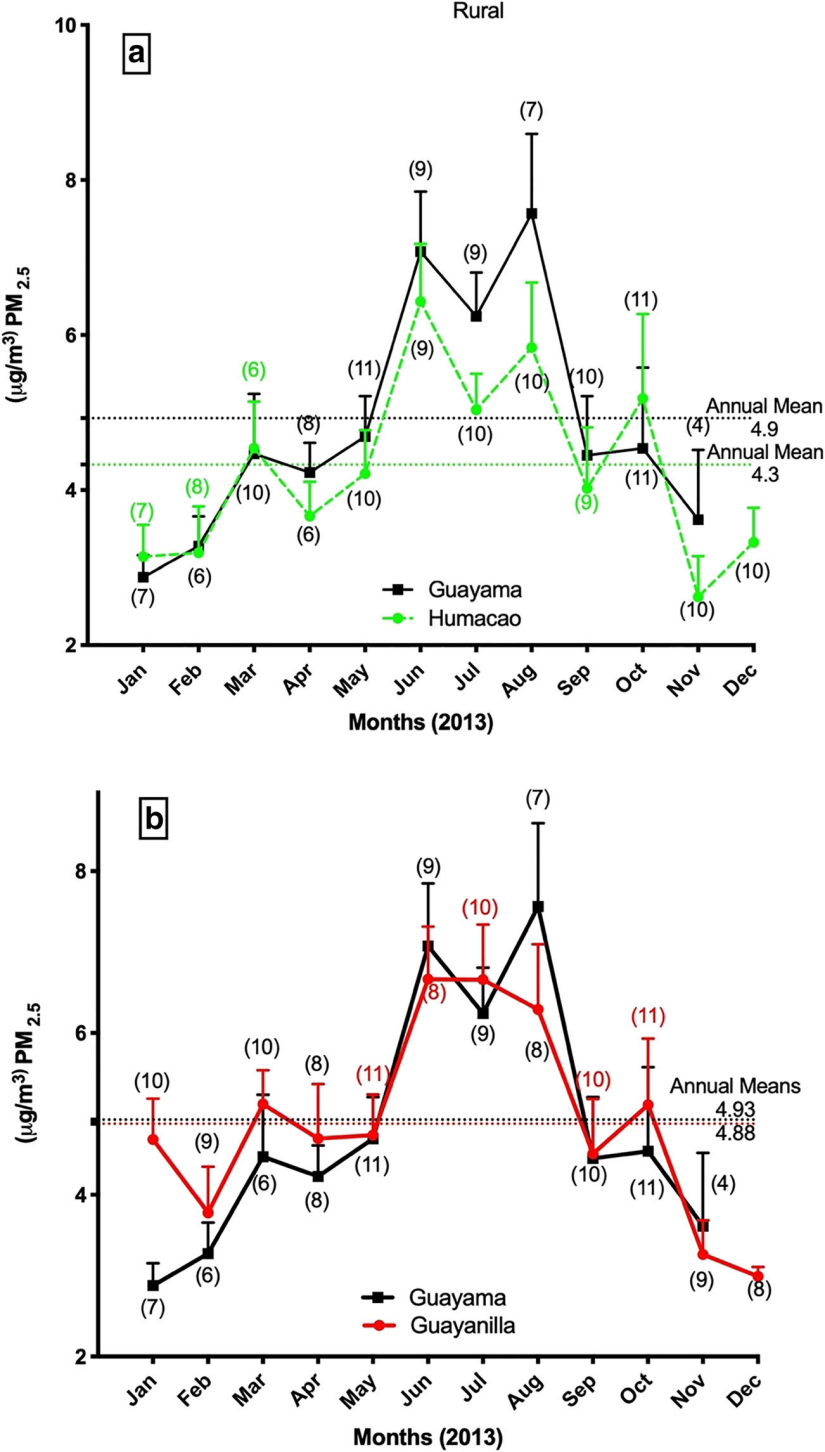
PM_2.5_ average monthly concentrations and annual means for rural sites Humacao, Guayama, and Guayanilla during 2013. The number of PM_2.5_ measurements is shown in parenthesis under or over each mean monthly value. Error bars are shown as the standard deviation of each month

**Fig. 4:**
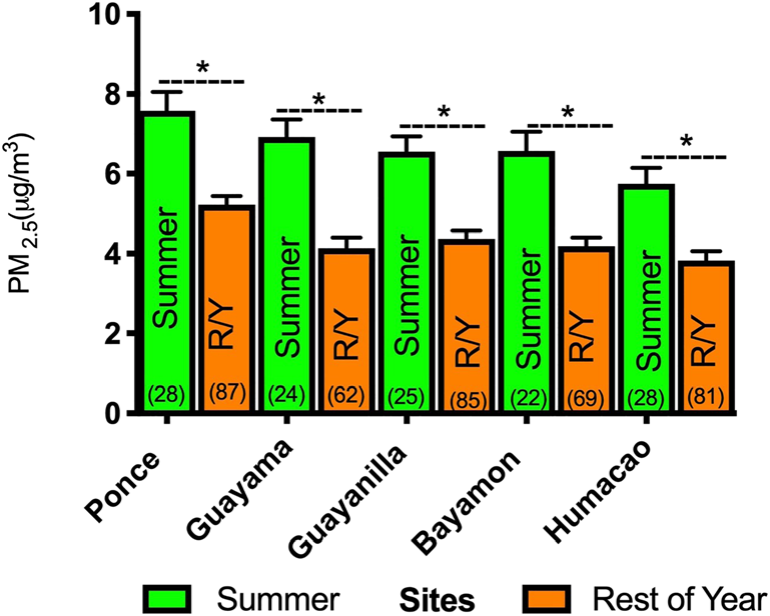
PM_2.5_ average concentrations during the summer and the rest of the year (R/Y) in 2013. Each bar represents the mean value with the standard error of the mean for summer PM_2.5_ in microgram per cubic meter compared to the mean PM_2.5_ value for the rest of the year not including summer. The asterisk represents a significant difference between summer and the rest of the year at a *p* ≤ 0.0001 (CI: 95%, *α* = 0.05). *n* values are shown in parenthesis

The climate in the southern areas of Puerto Rico is characterized by relatively high temperatures. The highest temperature was recorded from June to September (89.5 to 90.65 °F), while the relative humidity ranged from 72.00 to 80.30%. The prevailing wind directions were E/SE (4.2 and 1.4%) and NE (7.1 and 1.4%). The overall wind speed was an average of 5.7 mph. Bayamón (north) and Ponce (south) were among the municipalities covering more than 50% of the manufacturing operations according to the manufacturing industry census of Puerto Rico (2015). The annual average PM_2.5_ concentrations are shown in Figs. 2 and 3. A total of 33 days of African dust storm events were reported in 2013. The highest PM_2.5_ concentration was registered in Ponce (7.58 μg m^−3^) followed by Guayama (6.92 μg m^−3^), Bayamón (6.57 μg m^−3^), Guayanilla (6.5 μg m^−3^), and Humacao (5.76 μg m^−3^). All of the summer PM_2.5_ averages were significantly higher (*p* < 0.0001) when compared to the rest of the year at each municipality (Fig. 4).

The annual PM_2.5_ concentration at the Ponce urban site was significantly higher than those of any of the other rural and urban (Bayamón) sites, with an approximate mean of 4.69 ± 0.41 μg m^−3^, while Ponce had an average of 5.82 ± 0.22 μg m^−3^. Guayama and Guayanilla followed with an annual mean concentration of 4.93 ± 1.20 μg m^−3^ and 4.88 ± 1.50 μg m^−3^ respectively. Humacao’s annual PM_2.5_ concentration mean was the lowest of all the concentrations (4.33 ± 0.22 μg m^−3^) recorded for the 12 months.

### Trace metals in airborne particulate matter

**Fig. 5:**
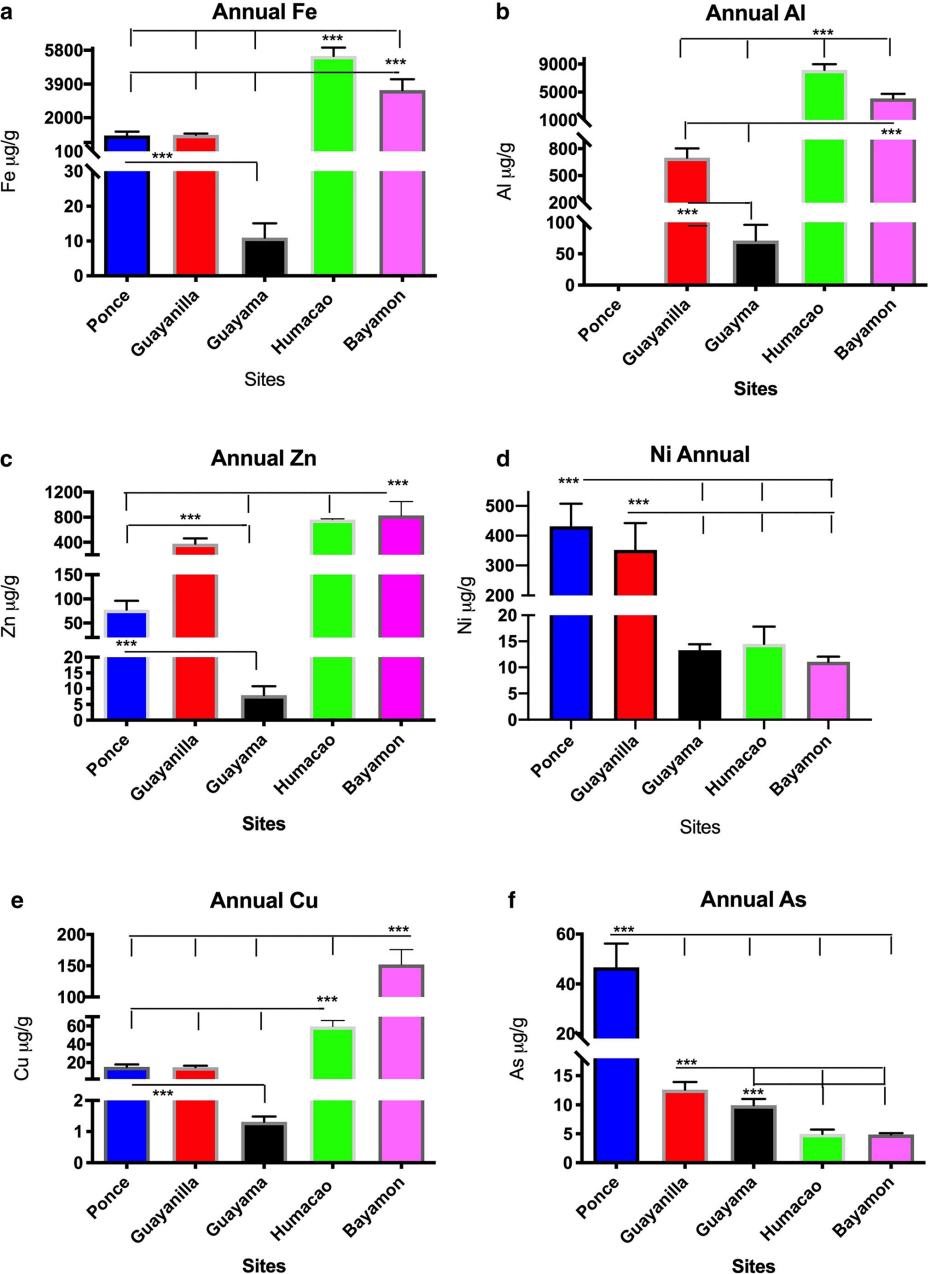
Distribution of toxic metals found in the highest comparable concentrations among sampling sites. Each bar represents the mean value with the standard error of the mean for each toxic metal in microgram per gram for each sampling site. The asterisk represents significant differences between sites (CI: 95%, *α* = 0.05)

**Fig. 6:**
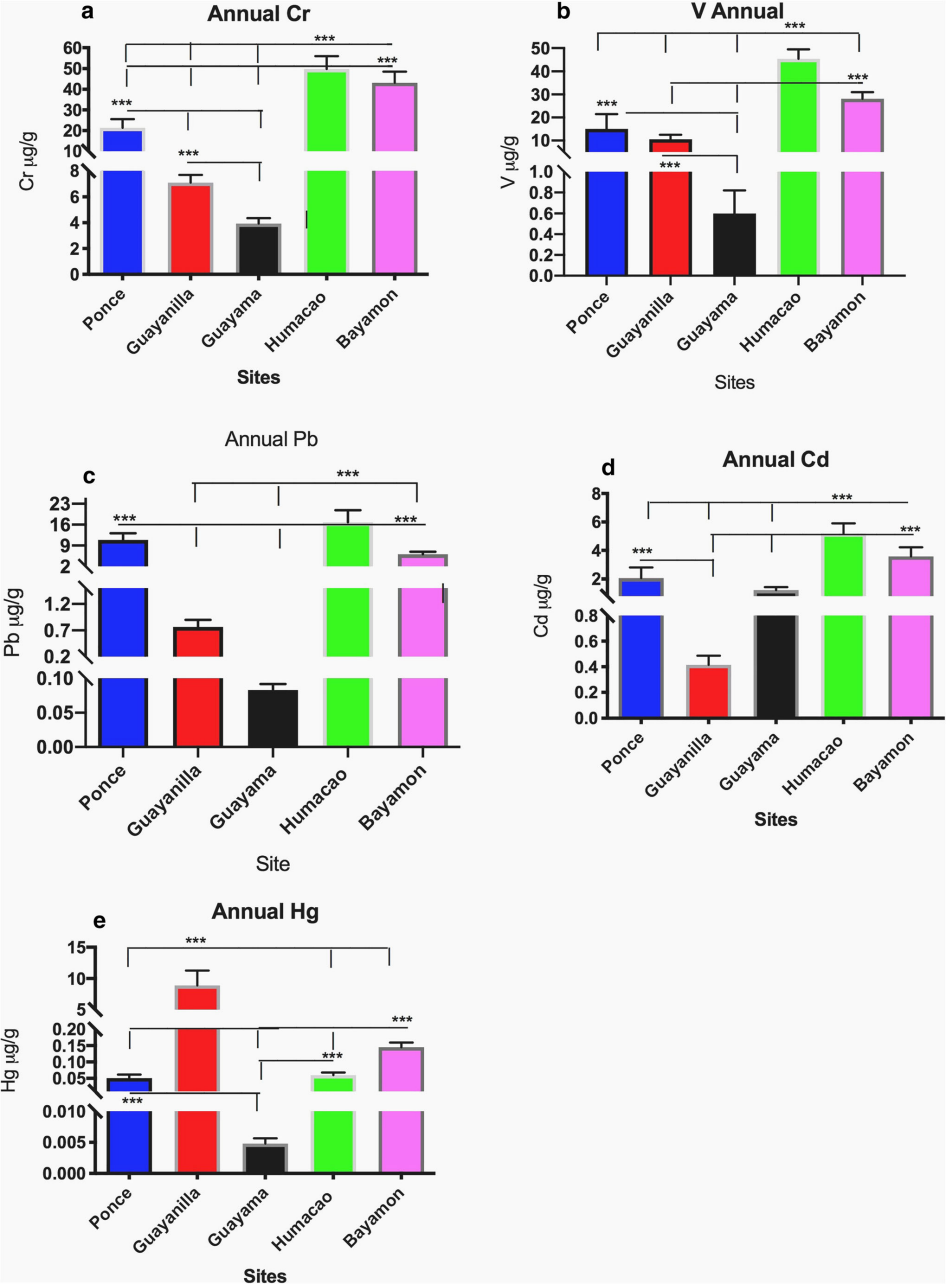
Toxic metal annual distribution in airborne PM_2.5_ among the different sampling sites. Each bar represents the mean value with the standard error of the mean for each toxic metal in microgram per gram for each sampling site annualized. The asterisk represents significant differences between sites (CI: 95%, *α* = 0.05)

To evaluate the relative air pollution at the five municipalities, we obtained and compared the PM_2.5_ extracts at all sites (Figs. 5–6). The differences in the toxic metal concentration by distribution are illustrated between the sites. Bayamón had the greatest concentrations of Cu and Zn, followed closely by the highly toxic metal concentrations of Al, Pb, V, Cd, Fe, and Cr found in Humacao. Although Humacao is considered a rural area, its PM_2.5_ composition contained the highest levels of the aforementioned six toxic metals followed closely by Bayamón. The other urban site (Ponce) was rich in As and Ni, followed by Guayanilla which is located downwind west from Ponce. The Guayanilla extract was the richest in Hg followed by Bayamón. The relative abundance of Hg in the extract was encountered during the period from January to May. The annual level of Hg in the Guayanilla PM_2.5_ extract was significantly higher than any of the other sites studied. Nevertheless, Hg was found to be present at all sites in airborne PM_2.5_ extracts (Fig. 6e). The lowest Hg levels were found in the municipality of Guayama. The Guayama PM_2.5_ extract was characterized by low levels of many toxic metals when compared to the other municipalities studied (Figs. 5–6).

Conversely, Guayama’s extract was characterized by the relatively high levels of As, Ni, Zn, and Cd. Although Guayama is considered a rural area, it is influenced by different anthropogenic sources (electric power plant and a coal ash plant). Bayamón and Humacao are similar in the sense that they both have relatively high concentrations of Fe, Al, Zn, Cu (Fig. 5a–c and e), Cd, V, Cr, and Pb (Fig. 6a–d). Ponce and Guayanilla showed elevated levels of Ni, As (Fig. 5d and f), Pb, and Hg (Fig. 6c and e). Also, Guayanilla was characterized by high levels of Ni, Zn (Fig. 5c and d), and Hg (Fig. 6e). The Zn concentrations predominantly found in the PM_2.5_ extracts from Bayamón, Humacao, and Guayanilla were significantly different from those from Ponce and Guayama (Fig. 5c). In summary, Guayanilla exhibited the highest level of Hg, Bayamón the highest Zn and Cu; Ponce, Ni and As; while Humacao, Fe, Al, Cr, V, Cd, and Pb. Many toxic metals were found in Humacao and are closely related to the levels in Bayamón.

Atmospheric toxic metal concentrations throughout the year are higher in urban sites compared to those of rural sites. The highest annual average concentrations were observed in Bayamón and Humacao sites (Al ranged from 18.60 to 16.30 ng m^−3^respectively), while not measured in Ponce, and the lowest concentration in Guayama (0.34 ng m^−3^). These concentrations need to be adjusted considering the extraction efficiency of the method employed (see the “Discussion” section). The levels of Zn in ambient air were the highest in Bayamón and Humacao (5.30–4.60 ng m^−3^ respectively) followed by Guayanilla (1.90 ng m^−3^) and Ponce; respectively, the lowest concentrations found were in Guayama (0.04 ng m^−3^). The highest levels of Ni in the air were characteristic of Ponce with an annual average of 2.50 ng m^−3^ followed by Guayanilla and Guayama (0.17 and 0.07 ng m^−3^ respectively). This presents supporting evidence that Ni is a toxic metal characteristic of the southern region. Similarly, the highest concentrations of ambient Hg were detected in Guayanilla (0.4 ng m^−3^) while the concentration of Hg in the rest of the sites was in the picogram per cubic meter range (Bayamón and Ponce followed). Therefore, Hg is also characteristic of the southern ambient air particles, particularly in hot spots. Another characteristic toxic metal in the south coast was As. The highest levels of As found in the sites studied in Puerto Rico ambient air were found in Ponce (0.30 ng m^−3^), while Guayanilla and Guayama exhibited values ≈ 0.06 ng m^−3^ and Bayamón and Humacao values of ≈ 0.02 ng m^−3^. Metals characteristic of the northern coast were Cu in Bayamón and Humacao sites with concentrations in the orders of 0.70 and 0.20 ng m^−3^ respectively, while the other southern stations ranged from 0.09 to 0.02 ng m^−3^ (Ponce, Guayanilla, and Guayama). Concentrations of chromium also found in Bayamón and Humacao were very similar in air ≈ 0.18 ng m^−3^, followed by Ponce (0.13 ng m^−3^) and Guayanilla and Guayama (0.04 and 0.02 ng m^−3^ respectively). Vanadium also had a similar concentration profile compared to Cr, with the highest concentrations in the air found in Bayamón and Humacao (0.12 and 0.15 ng m^−3^ respectively), followed by Ponce (0.1 ng m^−3^), and thereafter by Guayanilla and Guayama (0.05 and 0.02 ng m^−3^ respectively). Concentrations of Cd and Pb were very similar between all stations. Therefore, we describe Cr as to be at higher concentrations and thus associated with urban activities.

### Relative toxicity between urban and rural sites

BEAS-2B cell cultures were exposed to the annual PM_2.5_ from our different sampling sites for 24 h, and cell viability was then measured using the MTT assay. Likewise, the cell cultures were exposed to the vehicle only (0.01% DMSO) and a positive control (Triton X-100) (Fig. 8). The results indicate that the urban inorganic PM_2.5_ exposure had a stronger relative cytotoxic effect than the rural. The Bayamón PM_2.5_ extract capped at 25 μg/mL killing almost 15.87 ± 0.06% of cultured cells upon exposure. A concentration higher than 75 μg/mL of extract resulted in more than 79.90 ± 0.03% cells killed. We have calculated the LC_50_ for the urban PM_2.5_ extract from Bayamón to be 44.71 μg/mL. In comparison with the rural site of Humacao (the site closest to Bayamón), a similar concentration of 25 μg/mL did not show any relative toxicity, while the cell viability was comparable to the control. A higher concentration of this extract (50 μg/mL) killed almost 31.79 ± 0.52% of the cultured cells. The LC_50_ for the inorganic PM_2.5_ extract from Humacao was 68.89 μg/mL. However, when we obtain the relative toxicity of the other airborne PM extracts, we determined that Guayama (LC_50_ 43.5 μg/mL) and Bayamón (44.71 μg/mL) have comparable LC_50_ exhibiting similar toxic responses on human lung epithelial cells. These two sites comprise airborne PM_2.5_ that generates the most toxic response among all the sites studied. Guayanilla and Humacao follow with also close LC_50s_ of 52.7 μg/mL and 68.89 μg/mL respectively*.* Finally, Ponce (urban site) in the south coast includes airborne PM_2.5_ which generates the least toxic effect with the highest LC_50_ (78.5 μg/mL). There were large differences in the toxicity between (Ponce and Humacao) when compared to Bayamón, Guayama, and Guayanilla (Fig. 8). It is interesting to notice that there are considerably large differences between urban sites (north and south). The northern urban site generates more toxic airborne PM_2.5_ than its counterpart in the south. Ponce (the southern urban site) was not as toxic as our northern counterpart Bayamón, as an exposure of 100 μg/mL, saw a decrease of 36.49 ± 0.01% in cultured cells. Instead, the rural site of Guayama had a higher cytotoxic effect from the inorganic particles extract, with a 100-μg/mL exposure decreasing their survivability by 38.21 ± 0.08%. Guayanilla (a rural site) shows more toxicity than its closest urban site of Ponce.

## Discussion

Here, we report the measurement of 59 trace elements found in airborne PM_2.5_ extracts from five different sites in Puerto Rico, selected by their location (rural (south) and urban (north and south), during the 12 months in 2013. The samples were selected at random from a previously sampled pool granted by the Environmental Quality Board of Puerto Rico. This assessment provides a first-of-its-kind insight into the inorganic constituents of PM_2.5_ in the northeastern and southern regions of Puerto Rico, classifying the sample sites as rural or urban locations. Besides, it provides the basis for an initial comparison of PM_2.5_ in the Caribbean region.

Bayamón and Ponce (both urban sites) are considered among the most developed areas with industrial growth in Puerto Rico. Hence, the enrichment of particle pollution increases due to elevated anthropogenic activity. Humacao (rural), located in the east of the Island, is considered to have low industrial activity, most of which is related to the pharmaceutical industry. Humacao receives most of its airborne particulate from the reposition and deposition from the North Atlantic trade winds and the Caribbean Sea including local natural and anthropogenic sources. This study also includes PM_2.5_ from the municipalities of Guayama and Guayanilla (South Coast, Fig. 1 for relative locations of municipalities). Characteristic to the Caribbean islands, the trade winds are enriched with the African dust storms (Saharan Dust Storms) during June through August and some influence during the winter. The effects of the African dust storms are recorded with an increase in PM_2.5_ mass at all of the five municipalities during these 3 months (June-August; Figs. 2 and 3). Humacao and Guayanilla are both relatively low in industrial development compared to the other municipalities. We considered these specific locations to further incorporate metals most likely to be found at higher concentrations in anthropogenic and/or natural local sources, including those generated by industrial and domestic activities. We further evaluate the toxic metal distribution in PM_2.5_ of five locations throughout the Island. We recognize and acknowledge the contribution to toxic metals from volcanic ash and African dust storms. In 2013, the eruption of the Soufrière Hills volcano at Montserrat Island in the Caribbean released ash particles on and off until 2015. Events of Soufrière Hills Volcanic activity were recorded from January to May 2013 (20 days of activity) of which 4 days of ash fallings were reported for Puerto Rico.

**Fig. 7:**
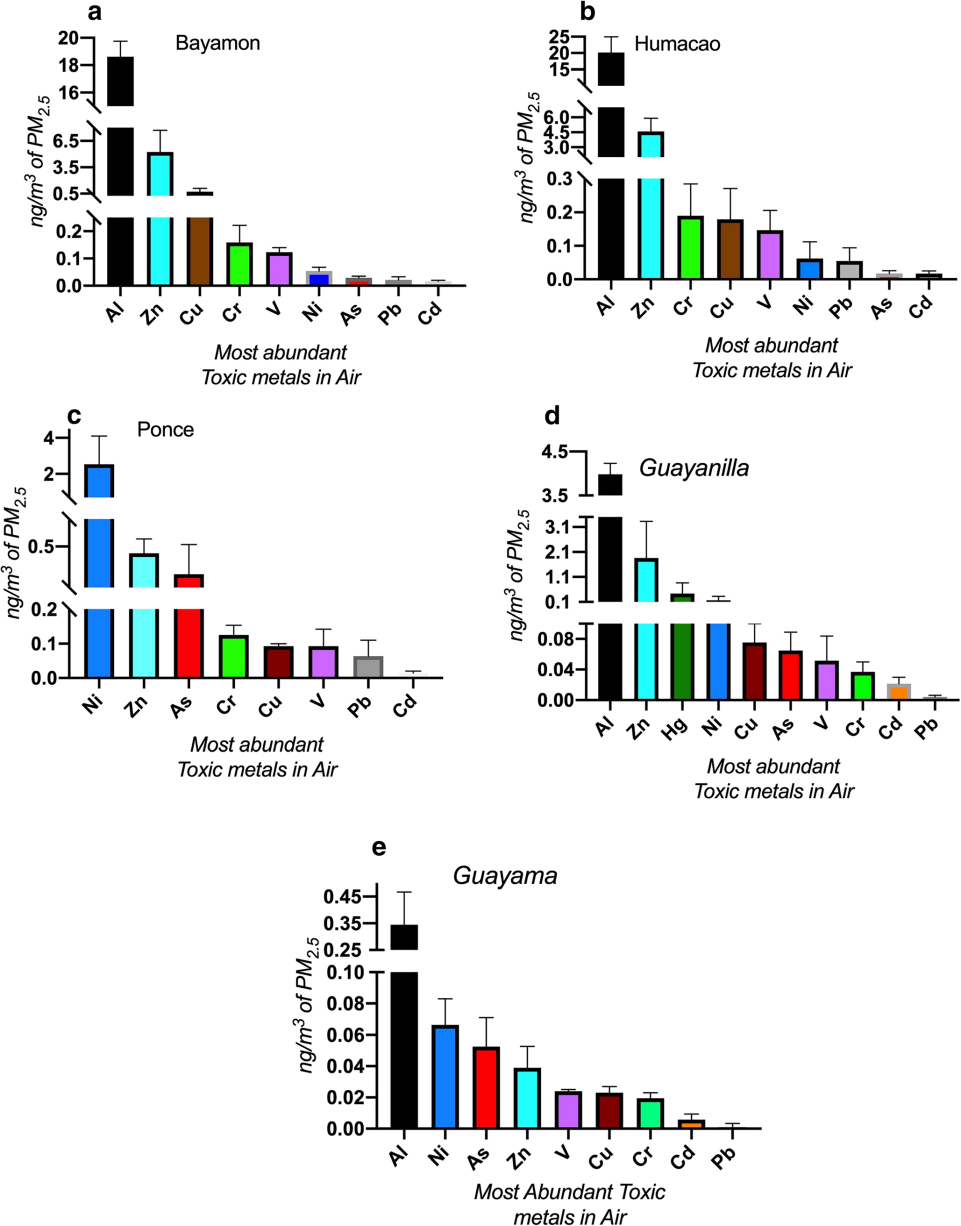
Toxic metal abundance in airborne PM_2.5_ at each of the five municipalities studied. Each bar represents the mean value with the standard error of the mean for each toxic metal in nanogram per cubic meter

Our findings support the idea of elevated toxic metals at the urban metropolitan site (Bayamón) compared to the other sites throughout the island of Puerto Rico, particularly more rural areas like Humacao, Guayama, and Guayanilla. As much as 1728 pounds of several toxic metals (Cr, Cu, Pb, Hg, Ni, and Zn) were released into the ambient air in Guayama by just one company in 2013 (EPA Toxic Release Inventory (TRI)). Incidentally, the ambient air from Guayama represents one of the most toxic extracts evaluated (LC_50_ 43.5 μg/mL). Some of these toxic metals, such as Ni and V, are products originating from fossil fuel combustion, reported in the air samples monitored during the year (Fig. 7). We also need to emphasize that the metal concentrations reported herein are underestimated since they are not normalized considering the extraction efficiency, which could represent as much as twice the reported concentration.

Following the definition of what is considered a toxic metal (a metallic element with a high atomic number and a specific gravity greater than 5.0), we choose to focus on the most commonly associated with industrial activity. The most abundant toxic metals at the different sites are shown and summarized in Fig. 7; here, Fe and Al are the most abundant in the environment and also found in the ambient air of Puerto Rico regardless of its location. The occurrence of toxic metals in the atmosphere and their distribution pattern in the study areas suggest that some of the available metals were transported via trade winds as aerosols to the urban and rural sites. We saw that V, Al, Pb, and Fe are found at higher concentrations in the rural sites. Of all the trace metals examined, Cu and Zn were significantly higher in the urban sites. High deposition of Zn could indicate possible sources such as in soil dust and strong emissions from anthropogenic sources (fossil fuel and biomass burning) and also attributed to elevated concentrations of these metals in the atmosphere around the study areas (Kyzioł 2002). Another study reported the presence of Zn in the ambient air due to the traffic emission originating from engine oils, brake, and exhaust systems (Bender and Lange 2001). This evidence provides an additional perspective for source-oriented enrichment of PM_2.5_ in both rural and urban areas.

Considering the differences among the distribution of toxic metals based on location, we found that V, Al, Pb, and Fe were higher at the rural sites compared to the urban sites; meanwhile, Cu and Zn, which are known to be vastly deposited in more urbanized regions due to fossil fuel combustion and biomass burning, were found to be elevated at the urban sites. It appears that the main reason for the high concentration levels of the toxic metals studied are the various sources of emission previously discussed in our study areas. Likewise, trade winds transporting African dust also contribute to the overall load of higher metal concentrations during the periods of May–September (Fig. 4).

**Fig. 8:**
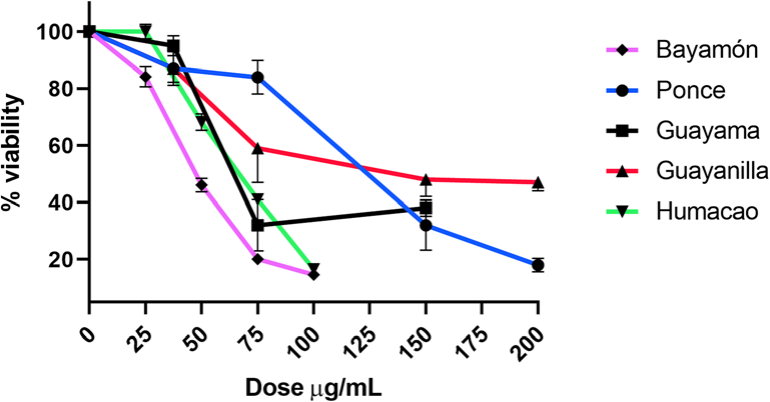
BEAS-2B dose-response curves after exposure to PM_2.5_. Each point represents the mean % (*n* = 3) viability, and error bars represent the SEM

Evaluating the toxic potential of the PM_2.5_ extract’s inorganic constituents, both rural and urban sites in the E/NE region of Puerto Rico had the highest toxic potential (Bayamón, Humacao, Guayama) compared to the southern region. This is could very well be associated with an earlier deposition of particles (E/NE) transported by the trade winds. In terms of the relative toxicity of PM_2.5_ inorganic extracts, the urban site of Bayamón and rural site of Humacao and Guayama had the most significant cytotoxic effect, with an LC_50_ difference of 35.10% for the average toxic response. Considering the urban and rural sites in the south region, there is a distinguishable trend towards a higher geological toxic potential increasing to the northeast. Guayama’s PM_2.5_ toxicity closely resembles that of Bayamón’s, compared to the other southern sites, followed by Ponce and the least toxic being Guayanilla (Fig. 8). The data shows a gradient increasing towards the northeastern regions. The trade winds effect could be potentiating this amplification in the toxic response, given that the sites in the northeast region are more exposed to the deposition and reposition of these events, compared to those located in the south and west. This sets the basis for conducting epidemiological studies to evaluate respiratory and cardiac disease distribution at various municipalities in Puerto Rico. Even though the treatments of bronchial epithelial cells are above the PM_2.5_ concentrations to which humans are exposed to, these extracts do not consider the coarse particulate fraction found in PM_2.5_. The concentrations to which we are exposed could be pari passu to the ones used as treatments in this study. PM_2.5_ constituents are known to be inducers of oxidative stress, shown by a significant reduction in the intracellular GSH concentrations and an increase in the expression of antioxidizing enzymes (Palleschi et al. 2018).

The results of this study show that the toxic metals associated with industrial activity, particularly biopharmaceuticals, contribute to the enrichment of the local particle pollution in the Puerto Rico environment. Although this seems to be the case, addressing our previous study and contrasting the trace metal content, there has been a considerable decrease in airborne toxic metal content in Puerto Rico (Molinelli et al. 2006). Technological advances have not only increased the efficiency and maximization of their intended output but also have improved by limiting the unintended byproducts that throughout history have been a primary source of contamination to our environment. Nevertheless, airborne pollution has had a global impact with many collaborative efforts been enforced to improve the quality of the air we breathe. This study provides a much-needed insight into the toxic content and air quality in Puerto Rico. Even though the toxic metal content does not compare to that of highly populated countries and cities, it is worrisome that it encompasses a comparable proportion to those shown in Table 1. Even considering our underestimated values (even if they were twice as high), we are still far below what is reported in other countries. Likewise, the concentrations determined for each sample and their respective inorganic constituents are understated given that we report only a fraction of PM_2.5_ as previously stated. Furthermore, this study did not consider organic pollutants, which constitute another source of exposure to humans. There were some limitations to this study given the lack of available air filter samples provided by the PR-EQB due to distribution with other proyect. However these samples are valuable since after  Hurricane Maria September of 2017 all filter samples at the agency’s storage bank were lost. This adds to the importance and significance of our work since similar information will not be available regarding trace metal concentrations on the island of Puerto Rico before those years. We believe these results will be the beginning of major studies regarding the change of particle pollution in the Puerto Rico environment before the events of Hurricane Maria, The Great Earthquake of January 7, 2020, and how anthropogenic pollution changed during the COVID-19 crisis.

**Table 1:** Comparison of airborne trace metal concentrations in different geographical world locations

Location	As	Cd	Cu	Fe	Ni	Pb	V	Zn	Ref.
China (ng m^−3^)	21.88	9.35	48.77	1317.20	8.54	189.98	7.29	418.27	(Wang et al. 2018)
Los Angeles, USA (ng m^−3^)	-	22.00	-	-	12.00	34.00	1.60	26.50	(Ljubimova et al. 2018)
IN, USA (ng m^−3^)	-	-	30.20	30,091,300.00	-	-	68,900.00	202,200.00	(Dietrich et al. 2019)
Greece (ng m^−3^)	5.78	0.58	-	-	2.19	10.40	-	-	(Vasilakos et al. 2007)
Targoviste City, Romania (ng m^−3^)	-	0.16	-	4.05	0.83	1.81	-	-	(Dunea et al. 2016)
Avonmouth, UK (ng m^−3^)	0.63	0.31	15.90	-	3.65	11.20	1.25	48.90	Environmental Agency (2014)
Peru (ng m^−3^)	32.00	14.00	184.00	10,161.00	38.00	242.00	25.00	1471.00	(De La Cruz et al. 2019)
Mexico City (ng m^−3^)	-	-	170.00	3800.00	70.00	480.00	50.00	1050.00	(Mugica et al. 2009)
Brisbane (ng m^−3^)	-	-	2.00	39.00	1.00	5.00	< 4.00	18.00	Hawas et al. (2003)
Sydney (ng m^−3^)	-	-	2.00	52.00	< 1.00	4.00	< 4.00	18.00	Hawas et al. (2003)
Melbourne (ng m^−3^)	-	-	4.00	76.00	1.00	4.00	< 4.00	8.00	Hawas et al. (2003)
Bayamón, Puerto Rico (ng m^−3^)	0.02	0.02	0.7	17	0.05	0.03	0.13	5.25	This work

## Data Availability

The datasets from which the current study was created are available from the corresponding author on reasonable request.
